# Intracellular and extracellular targets as mechanisms of cancer therapy by nanomaterials in relation to their physicochemical properties

**DOI:** 10.1002/wnan.1680

**Published:** 2020-10-27

**Authors:** Charlene Andraos, Mary Gulumian

**Affiliations:** ^1^ Toxicology Department National Institute for Occupational Health Johannesburg South Africa; ^2^ Haematology and Molecular Medicine Department University of the Witwatersrand Johannesburg South Africa; ^3^ Water Research Group, Unit for Environmental Sciences and Management North West University Potchefstroom South Africa

**Keywords:** cancer therapy, cell death pathways, intracellular mechanisms, nanomaterial characteristics, nanomedicine

## Abstract

Cancer nanomedicine has evolved in recent years and is only expected to increase due to the ease with which nanomaterials (NMs) may be manipulated to the advantage of the cancer patient. The success of nanomedicine is dependent on the cell death mechanism, which in turn is dependent on the organelle initially targeted. The success of cancer nanomedicine is also dependent on other cellular mechanisms such as the induction of autophagy dysfunction, manipulation of the tumor microenvironment (TME) and secretome or induction of host immune responses. Current cancer phototherapies for example, photothermal‐ or photodynamic therapies as well as radio enhancement also form a major part of cancer nanomedicine. In general, cancer nanomedicine may be grouped into those NMs exhibiting inherent anti‐cancer properties that is, self‐therapeutic NMs (Group 1), NMs leading to localization of phototherapies or radio‐enhancement (Group 2), and NMs as nanocarriers in the absence or presence of external radiation (Group 3). The recent advances of these three groups, together with their advantages and disadvantages as well as their cellular mechanisms and ultimate outcomes are summarized in this review. By exploiting these different intracellular mechanisms involved in initiating cell death pathways, it is possible to synthesize NMs that may have the desirable characteristics to maximize their efficacy in cancer therapy. Therefore, a summary of these important physicochemical characteristics is also presented that need to be considered for optimal cancer cell targeting and initiation of mechanisms that will lead to cancerous cell death.

This article is categorized under:

Therapeutic Approaches and Drug Discovery > Nanomedicine for Oncologic Disease
Toxicology and Regulatory Issues in Nanomedicine > Toxicology of Nanomaterials
Toxicology and Regulatory Issues in Nanomedicine > Regulatory and Policy Issues in Nanomedicine

## Graphical abstract

Cellular mechanisms exploited in cancer nanomedicine.

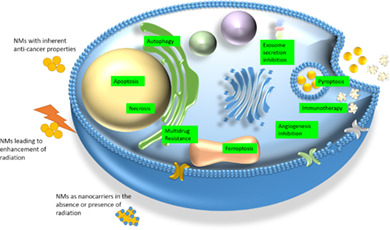

## 1 INTRODUCTION

The advances in nanomedicine have gained much attention in the last two decades based on the benefits of nanomaterials (NMs) over those of traditional chemotherapeutics. One of the most important advantages of NMs is their ability to target tumor tissue via the enhanced permeability and retention (EPR) effect. During EPR, NMs passively enter into the characteristic permeable vessels of tumor tissue and is then effectively retained (Greish, 2010). NMs may also be targeted actively to tumor cells by linking high‐affinity ligands to their modifiable surfaces. The specificity of NMs thereby spares healthy tissues from treatment‐induced side effects. NMs also allow successful transportation of chemo drugs to tumor sites via linking or encapsulation within their structures. Once at the tumor site, the NM may initiate cell killing through the assistance of an externally applied radiation source that results in heat production (i.e., photothermal therapy, PTT), reactive oxygen species (ROS) production (i.e., photodynamic therapy; PDT), or radio enhancement. NMs themselves, in the absence of chemo drugs or externally applied radiation may also exhibit intrinsic anti‐cancer properties. Nanomedicines are often divided into those that are organic (liposomes polymeric micelles, dendrimers, polymeric NPs, and polymer drug conjugates) and those that are inorganic (NMs consisting of metals, metal‐oxides, carbon, quantum dots (QDs), silica, etc.; Adeel, Duzagac, Canzonieri, & Rizzolio, 2020; Navya et al., 2019; Q. Zhou, Zhang, & Wu, 2017). For the purposes of this review, only inorganic NMs are considered.

Various cellular mechanisms may be initiated by NMs to lead to toxicity or inhibition of tumor growth, irrespective of whether the NM acts on its own or in combination with a chemo drug and/or externally applied radiation. One of the hallmarks of cancer is its ability to evade cell death by adaptation of its intracellular mechanisms that allow it to thrive in stress conditions including hypoxia and nutrient deficiency (Fouad & Aanei, 2017; Wlodkowic, Skommer, McGuinness, Hillier, & Darzynkiewicz, 2009). Therefore, researchers argue that initiating necrosis is more desirable in cancer therapy as cancer cells often adapt to controlled cell death pathways such as apoptosis (Piao & Amaravadi, 2016). Other researchers argue that necrosis follows a disorderly pattern and is not as controlled as apoptosis. Necrosis also produces an inflammatory response, which might be beneficial for treatment in some cases (Spyratou, Makropoulou, Efstathopoulos, Georgakilas, & Sihver, 2017) or undesirable in other cases (Hainan Sun, Jia, Jiang, & Zhai, 2018; E. Zhang et al., 2014). Cancer cells also show greater dependence on autophagy as a pro‐survival mechanism in stressful conditions. Although autophagy is not considered a cell death mechanism, it has been proposed that modulation of the autophagic pathway in cancer cells may initiate cell death pathways or sensitize them to therapy. Ferroptosis on the other hand is a novel cell death mechanism with great potential in cancer therapy as it is initiated by intracellular iron metabolism alterations. Moreover, cancer cells are known to exhibit an increased iron demand for survival thereby making ferroptosis an attractive mechanism to target (Hassannia, Vandenabeele, & Berghe, 2019). Pyroptosis as a cell death mechanism in cancer therapy is also still in its infancy and the initiation of pyroptosis by NMs in cancer cell models is limited.

In this review, cell death mechanisms involved in cancer nanomedicine are discussed in terms of the following:

1. NMs exhibiting inherent anti‐cancer properties.
2. NMs leading to localization of radiation for PTT‐, PDT‐, or radio‐enhancement.
3. NMs as nanocarriers in the absence or presence of radiation.

In addition, mechanisms leading to inhibition of angiogenesis, epithelial–mesenchymal transition (EMT), tumor exosome release, multidrug resistance (MDR), and cancer immunotherapy are also considered. By understanding these cellular mechanisms involved in cancer nanomedicine, it will be possible to utilize the most appropriate strategy for cancer eradication and allow future nanomedicine development.

## 2 NMs EXHIBITING INHERENT ANTI‐CANCER PROPERTIES

NMs alone could exhibit intrinsic anti‐cancer characteristics, in the absence of other therapeutic compounds and/or additional external radiation. These “self‐therapeutic” NMs therefore have the advantage of being less complex and carry fewer side effects (Adeel et al., 2020). The following sections discuss the cellular mechanisms in relation to their intracellular organelle localization, which are involved in inorganic “self‐therapeutic” NMs.

### 2.1 Nucleus

The nucleus is the center that controls cell growth, proliferation, apoptosis, metabolism, gene activation and cell cycle management (L. Pan, Liu, & Shi, 2018). It is therefore one of the most targeted organelles in cancer nanomedicine. However, nuclear targeting is made complicated by the nuclear pore complex (NPC), which is a porous double layered nuclear envelope separating the nucleus from the cytosol. Consequently, several strategies have been employed to increase NM nuclear internalization (L. Pan et al., 2018). NMs may enter the nucleus passively depending on their physicochemical characteristics. For example, AuNPs of 2, 6, and 8.5 nm enter through the NPCs passively via Brownian motion (Huo et al., 2014; Qiu et al., 2015). Larger particles between 10 and 50 nm enter through the NPCs actively while those larger than 50 nm have been shown to enter by physically opening the nuclear envelope that is, Prussian blue NPs and Au nanorods (AuNRs; 50–100 nm). Clearly, for nuclear targeting, smaller NMs appear to be more effective. Indeed, large silver NPs (AgNPs) of 53.8 and 137.3 nm and AuNPs of 86 nm had minimal to no effect on nuclear‐targeted therapy compared to their smaller counterparts (Kodiha et al., 2014; L. Liu et al., 2011). In addition to size, the shape of NMs is another important factor to consider. Hinde et al. (2017) demonstrated that the concentrations of rods (5–10 nm × 100–300 nm) and worm‐like (5–10 nm × 400–700 nm) copolymer NPs in the nucleus were significantly higher compared with low‐aspect‐ratio copolymer NPs that is, micelles (20 nm × 20 nm) and vesicles (100 nm × 100 nm). This was ascribed to the diameters of high‐aspect‐ratio rods and worms that are smaller than the central size of NPCs. NM charge was also important for nuclear entry. Ojea‐Jiménez, García‐Fernández, Lorenzo, and Puntes (2012) showed that 13 nm cationic AuNPs functionalized with ‐PEG‐NH^3+^ (10.0 mV) successfully accumulated in the nucleus of human fibroblast 1BR3G cells, whereas no cellular uptake was observed for anionic AuNPs functionalized with ‐PEG‐COO¯ (−41.5 mV).

The active nuclear targeting of NMs involves attaching nuclear localization signal (NLS) peptides, which are specifically recognized by the NPC and consists of positively‐charged lysine and arginine residues for enhanced nuclear uptake. Examples of successful NLSs include the simian virus SV 40 large T antigen, adenovirus, and HIV TAT peptide (Limin Pan, Liu, & Shi, 2017; L. Pan et al., 2018). NLS peptides are known to associate with karyopherins (importins) in the cytoplasm, after which translocation to the nucleus occurs via the importin a/b pathway (Kang, Mackey, & El‐Sayed, 2010; L. Pan et al., 2018). Nucleolin has been used as a trafficking molecule to transport ligands directly from the cell membrane to the nucleus in cancer cells as it is overexpressed in these cells (Dam, Lee et al., 2012). In addition to passive or active nuclear targeting, other mechanisms may also be used to allow nuclear internalization. For example, the induction of lysosomal destruction through ^1^O_2_ oxidation by 83.6 nm nanoplatforms may result in lipid peroxidation of the nuclear membrane, therefore allowing access into the nucleus (Zhu et al., 2018).

Several NMs have shown intrinsic characteristics allowing them to damage the nuclear environment directly. Kang et al. (2010) showed that 30 nm PEGylated (poly‐ethylene glycol stabilized) AuNPs liganded with NLS could selectively and directly form DNA double strand breaks (DSBs) as well as disturb the division of human oral squamous cell carcinoma cancer cells via cytokinesis arrest. This resulted in the failure of complete cell division at the mitotic phase and ultimately apoptosis. The exact mechanism of how the AuNPs induced DSBs and cytokinesis arrest was not studied further. However, NMs may induce DSBs via several mechanisms, ranging from NM‐induced oxidative stress on the DNA to the impairment of the DNA repair process by: (1) sequestration of the DNA repair enzymes within the NM protein corona or; (2) direct NM interaction with transcription factors and DNA repair enzymes (Carriere, Sauvaigo, Douki, & Ravanat, 2016). In addition to inducing DSBs and cell cycle arrest, NMs also appear to lead to conformational changes of the nucleus itself. For example, a 32 nm nanoconstruct consisting of gold nanostars (AuNSs) and a nucleolin DNA aptamer induced extreme deformation of the nuclear envelope through the formation of nuclear intruding folds, increased caspase‐3 and ‐7 activities and induced DSBs, ultimately inducing apoptosis (Dam, Culver et al., 2012; Dam, Lee, et al., 2012).

### 2.2 Mitochondria

Mitochondria are critical energy‐producing cellular organelles and decisive regulators of more than one cell death mechanism including the intrinsic pathway of apoptosis and the most recently identified ferroptosis.

For mitochondrial targeting, a larger range of NM sizes appears to internalize successfully into mitochondria. For example, sizes of NPs successfully internalized range from 15 nm iron oxide (Fe_3_O_4_) NPs (Jung et al., 2015) to 0.81 nm × 500–1,500 nm single‐walled carbon nanotubes (SWCNTs) (F. Zhou, Wu, Wu, Chen, & Xing, 2011; F. Zhou et al., 2010). Marrache and Dhar (2012) showed that, among NPs with sizes 80–330 nm, maximum uptake of NPs 80–100 nm in the mitochondria of HeLa cells was observed. On the other hand, NMs smaller than 10 nm have been shown to easily permeate through the mitochondrial membrane through voltage‐dependent anion channels (C.‐G. Liu, Han, Kankala, Wang, & Chen, 2020). NM charge is also important in mitochondrial targeting. Cancer cells exhibit a more negative mitochondrial potential (−150 to −180 mV) compared to normal cells with (up to ~60 mV potential difference (Z. Ma, Han, Dai, & Han, 2018; Marrache & Dhar, 2015). Marrache and Dhar (2015) and Marrache and Dhar (2012) showed that cationic NPs entered mitochondria, more so at ∼34 mV, compared to 1.3–22 mV, whereas anionic NPs between −23.7 and −12 mV are mostly distributed in the cytosol. Various mitochondrial‐specific ligands are also often utilized for active targeting. A well‐known mitochondrial ligand, lipophilic triphenyl phosphonium (TPP), easily permeates lipid bilayers due to their cationic nature (Battogtokh et al., 2018). Yang et al. (2015) demonstrated higher mitochondrial selectivity of 50 nm cationic TPP‐AuNPs in breast cancer cells compared to anionic AuNPs of similar size. Tu et al. (2018) demonstrated the successful internalization of mitochondrial‐targeted graphene nanosheets, which could carry a negative charge (−8 mV) at physiological pH and upon entering an acidic environment, the nanosheets became positive (+15 mV) due to the internal attack of the pH‐sensitive amide carbonyl group by β‐carboxylate, which consequently produced NH_3_^+^.

Ferroptosis is a novel, nonapoptotic programmed cell death mechanism showing promise in cancer therapy (M. Liu et al., 2019; Shen, Song, et al., 2018). Ferroptosis is characterized by ROS derived from iron metabolism via the Fenton reaction and GSH depletion via either glutathione peroxidase‐4 (GPX4) downregulation or depletion of cysteine, a precursor for GSH (M. Gao et al., 2019). GSH depletion results in an increase in the level of lipid peroxides, which in turn is further increased by the Fenton reaction and ROS generation. Whether the mitochondria plays an active role in suppressing or initiating ferroptosis induced by GPX4 inactivation and/or cysteine depletion is still under debate (M. Gao et al., 2019). Nevertheless, ferroptosis results in mitochondrial morphological alterations including smaller size, decreased, or vanishing mitochondria crista, condensed mitochondrial membrane, and ruptured outer mitochondrial membrane.

As iron is the key mediator in ferroptosis, it is conceivable that iron‐based NMs, for example Fe_3_O_4_ NPs, would play a role in ferroptosis therapy. The tumor environment is more acidic than that of normal tissue creating an ideal environment for the release of ferrous (Fe^2+^) or ferric (Fe^3+^) ions from NPs, which could induce ferroptosis. For example, Huang, Wei, Chiu, Wu, and Shieh (2019) demonstrated that 123.4 nm zero‐valent iron NPs induced ferroptosis, following autophagy, in oral cancer cells via the Fenton reaction. The latter in turn resulted in ROS production, mitochondrial lipid peroxidation, and reduced levels of GPX4. Other noniron NMs could also induce ferroptosis by sequestration of iron from the surrounding environment. For example, Kim et al. (2016) showed that 6 nm PEGylated silica NPs (SiNPs) functionalized with melanoma‐targeting peptides induced ferroptosis in amino acid‐starved cancer cells and inhibited tumor growth in cancer‐bearing mice. They suggested that the deprotonated surface silanol groups and/or fractal internal structure of the NP itself may lead to iron adsorption and loading onto cancer cells. This increased iron uptake could lead to GSH depletion due to increased ROS generation. Lin, Song, et al. (2018) showed that PEGylated MnO_2_‐coated thiol‐functionalized mesoporous SiNPs could induce ferroptosis in U87MG human glioma cells. The MnO_2_ shell could undergo a redox reaction with intracellular GSH to yield Mn^2+^ and GSSG thereby depleting GSH levels while at the same time transform endogenous H_2_O_2_ produced by the mitochondria into hydroxyl radicals via the Fenton reaction. Similarly, Wang, Li, Duan, et al. (2018) and Wang, Li, Qiao, et al. (2018) showed that 59 nm arginine‐rich manganese (Mn) silicate nanobubbles were able to induce ferroptosis by GSH depletion possibly via oxidation thereby decreasing GPX4 activity and expression. This resulted in oxidative stress, increased lipid peroxidation, and ultimately ferroptosis. These nanobubbles also showed a faster Mn^2+^ release rate than that of MnO‐PEG NPs, indicating that the nanobubble structure was more beneficial for ferroptosis‐induction in cancer cells.

### 2.3 Lysosome, autophagosome, and autophagolysosome

The lysosome forms an integral part of the cell death machinery so it is conceivable that it would be involved in multiple types of cellular mechanisms such as autophagy, apoptosis or necrosis (C.‐G. Liu et al., 2020; F. Wang, Gómez‐Sintes, & Boya, 2018). Indeed, it has been suggested that the effects of lysosomal membrane permeabilization (LMP) could be more detrimental to cancer cells than normal cells as cancer cells exhibit a higher availability of cathepsins (C.‐G. Liu et al., 2020). Lysosomal targeting depends greatly on the mechanism of entry of NMs, which in turn also depends on the NM size. For example, NMs of ~60 nm may easily be internalized by caveolin‐dependent endocytosis, during which the NM will end up in caveosomes. Larger NMs of ~120 nm may be internalized through clathrin‐dependent/independent endocytosis, which then depends on the NM surface topography as well as their shape. NMs that are internalized via endocytosis end up within lysosomes and either undergo degradation or enter the cytoplasm through lysosomal escape pathways (C.‐G. Liu et al., 2020). NMs may also be targeted directly and actively to lysosomal membrane markers, for example, lysosome‐associated membrane protein 1 (LAMP1), and so on (E. Zhang et al., 2014). The mechanism of entry of NMs is also dependent on NM surface topography. NPs with roughened surfaces showed an increased tendency to enter cells via pathways independent from lysosomes (X. Sun, Li, Yang, Jia, & Liu, 2018; W. Wang et al., 2017).

Several NMs show intrinsic abilities to induce LMP. AuNPs and AgNPs may lead to alkalization of the lysosomal interior and consequently induce LMP (X. Ma et al., 2011; Miyayama, Fujiki, & Matsuoka, 2018). Yang, Wu, Wang, Su, and Shieh (2019) showed that 28.8 nm silica‐modified zero‐valent iron NPs induced LMP via lysosomal Fe^3+^ burst thereby inducing LMP as well as both apoptosis and necrosis. Borkowska et al. (2020) showed that mixed‐charge 5 nm AuNPs could selectively target lysosomes in cancerous HT1080 fibrosarcoma cells resulting from distinct pH‐dependent aggregation events thereby resulting in the formation of large NP assemblies and crystals inside lysosomes. These assemblies cannot be cleared by exocytosis and therefore result in lysosomal swelling, LMP, impaired lysosomal functions and apoptosis induction. Using A459 lung cancer cells, Miyayama et al. (2018) showed that 60 nm AgNPs could reduce the expression of transcription factor EB (TFEB), which is a regulator of lysosomal biogenesis and autophagy. This resulted in lysosomal dysfunction and blockade of autophagic flux as well as accumulation of autophagosomes, as confirmed by increased levels of the autophagic markers microtubule‐associated protein 1 light chain 3B‐II (LC3B‐11) and p62. SiNPs were also shown to accumulate in the lysosomes of human cervix carcinoma HeLa cells resulting in the perturbation of autophagy‐mediated protein turnover and the accumulation of LC3‐ and p62‐positive autophagosomes (Schütz et al., 2016). Finally, Gao et al. (2014) could demonstrate the successful induction of LMP and apoptosis via ROS generation by Au‐zinc oxide (ZnO) nanohybrids, targeted to the lysosomes of human liver HepG2 cancer cells.

### 2.4 Golgi/endoplasmic reticulum (ER)

The Golgi is the central organelle of the cell secretory pathway, which interacts with the ER and carries out posttranslational modification of newly synthesized proteins (Sakhrani & Padh, 2013). The ER on the other hand, which is a cytosolic membrane‐bound network connected to the nucleus, mitochondria, and plasma membrane, facilitates the folding of secretory and membrane proteins, and is involved in calcium storage and signaling. The major cell death mechanism involved in Golgi‐ER targeting appears to be apoptosis as ER stress generally leads to mitochondrial‐induced apoptosis due to the close communication between the ER and the mitochondria (Boelens, Lust, Offner, Bracke, & Vanhoecke, 2007; Gupta et al., 2010; Sakhrani & Padh, 2013; Wlodkowic et al., 2009).

Using endometrial cancer JEC cells, Wang et al. (2015) showed that 5.48 nm realgar (A_S4_S_4_) quantum dots (QDs) demonstrated anti‐tumor effects, which was mediated through the ER stress signaling pathway ultimately resulting in mitochondrial‐mediated apoptosis and necrosis. The QD treatment resulted in widespread vacuolization, which is a characteristic morphological change associated with the distension of ER. In addition, apoptosis‐inducting ER stress proteins that is, immunoglobulin heavy‐chain‐binding protein GRP78 (aka BiP) and GADD153 (aka CHOP) was increased.

Not only is apoptosis the dominant mechanism in ER stress but ER‐induced autophagy may also be induced by NMs. For example, 86 nm SiNPs induced ER autophagy in human colon HCT‐116 cancer cells by increasing the quantity of LC3‐I to LC3‐II (Wei, Wang, Luo, Li, & Duan, 2017). Similarly, Wang, Li, Duan, et al. (2018) and Wang, Li, Qiao, et al. (2018) demonstrated that 57.7 nm SiNPs stimulated ROS generation, which in turn induced ER autophagy through activation of the EIF2AK3 and ATF6 pathways.

### 2.5 Multiple organelles

Certain NMs show intrinsic anti‐cancer activities through the destruction of more than one organelle. For example, Fe_3_O_4_ NPs initiated LMP and autophagosome accumulation in MCF‐7 human breast cancer cells and inhibited mitochondrial function via a decrease in ATP production. These NPs also inhibited the activity of the protein kinase mechanistic Target of Rapamycin (mTOR) and activated Unc‐51 Like Autophagy Activating Kinase 1 (ULK1), a key player in autophagy induction. Lastly, these NPs could physically disrupt both the ER and Golgi thereby further inducing autophagy. Therefore, severe autophagy induction through the destruction of multiple organelles by these Fe_3_O_4_ NPs ultimately resulted in apoptosis (X. Zhang et al., 2016). Similarly, Bai, Zhang, Zhang, Huang, and Gurunathan (2017) showed multiple organelle damage by 20 nm ZnO NPs, which induced both autophagy and apoptosis. These NPs could induce ROS generation and oxidative stress thereby disrupting the mitochondrial membrane potential. In addition, these ZnO NPs could induce DSBs, increase the expression of p53, LC3, Bax and caspase‐9, and decrease the expression of the anti‐apoptotic protein Bcl‐2. In another study using human hepatocellular carcinoma HepG2 cells, Mishra, Zheng, Tang, and Goering (2016) showed that polyvinylpyrrolidone (PVP)‐coated AgNPs (10–100 nm) led to apoptosis through increased LMP and autophagy induction via enhanced LC3B expression and activation of NLRP3‐inflammasomes via enhanced expression of caspase‐1 and IL‐1β. With these NPs apoptosis induction was also confirmed through the increased expression of the pro‐apoptotic protein caspase‐3. Finally, the AgNPs also induced apoptosis via the ER stress pathway by activating GADD153 expression. Similarly, Buttacavoli et al. (2018) showed that the anti‐cancer activity of Ag+ released from AgNPs was mainly associated with accumulation within the mitochondria and nucleus, which induced ER stress, oxidative stress, and mitochondrial impairment thereby inducing apoptosis.

It can be said that although not many studies have assessed the adverse effects of NMs on multiple organelles and their subsequent cell death mechanisms, these studies may indeed contribute in advancing nanomedicine as the effect of NMs is likely to affect more than one organelle and therefore exert their effects through multiple cellular mechanisms.

### 2.6 Angiogenesis and EMT

Angiogenesis, defined as the formation of new vascular networks, and EMT, defined as the differentiation of epithelial cells into motile mesenchymal cells, both play key roles in cancer progression and metastasis (Kargozar, Baino, Hamzehlou, Hamblin, & Mozafari, 2020; Lamouille, Xu, & Derynck, 2014; Nishida, Yano, Nishida, Kamura, & Kojiro, 2006). Thus, preventing angiogenesis and reversing EMT have shown promise in cancer nanomedicine. Heparin‐binding growth factors (HB‐GFs) play a major role in both angiogenesis and EMT. For example, the vascular endothelial growth factor (VEGF), in particular, has been shown to induce both angiogenesis and EMT through the modulation of expression of transcription factors for example, Snail and Twist (Lamouille et al., 2014). Other growth factors that play major roles in EMT include heparin‐binding EGF‐like growth factor (HB‐EGF), basic fibroblast GF (bFGF), transforming growth factor beta (TGF‐β) and tumor necrosis factor alpha (TNF‐α), among others, and have been suggested as targeting molecules to potentially reverse EMT (Arvizo et al., 2011; Arvizo et al., 2013).

AuNPs exhibit strong anti‐angiogenic properties and can directly inhibit the function of a multitude of HB–GFs such as VEGF165 and bFGF through the unfolding of the protein structure (Arvizo et al., 2011; Arvizo et al., 2013; Mukherjee et al., 2005). Using HUVEC and NIH3T3 cells and nude mice ear and mice ovarian tumors, Bhattacharya et al. (2004) and Mukherjee et al. (2005) showed that 5 nm AuNPs bind these growth factors via their heparin‐binding domains presumably through cysteine residues (Mukherjee et al., 2005). Binding of AuNPs to VEGF165 prevented their interaction with cell surface receptors (i.e., VEGFR‐2) thereby inhibiting receptor‐phosphorylation, a critical step in angiogenesis (Resham Bhattacharya & Mukherjee, 2008). AuNPs (5 nm) have also shown to result in cell cycle arrest of multiple myeloma cell lines by up‐regulating the cell cycle inhibitor proteins p21 and p27, not as a direct result of interaction of the AuNPs with cell cycle proteins but rather as a downstream consequence of AuNPs binding directly to VEGF (Resham Bhattacharya et al., 2007).

Arvizo et al. (2013) also showed that 20 nm AuNPs reversed EMT by up‐regulating E‐Cadherin (E‐Cad), and down‐regulating Snail, N‐Cadherin, and Vimentin in three ovarian cancer cell lines (A2780, OVCAR5, and SKOV3‐ip). E‐Cad repression is the hallmark of EMT and occurs through transcriptional repressors such as Vimentin, Snail, Slug, Twist, and so on. The AuNP treatment also resulted in the localization of E‐Cad from a perinuclear site to the cell membrane, further supporting EMT reversal. Arvizo et al. (2013) could also show that binding of secreted HB–GFs of ovarian cancer cell lines by AuNPs led to the reduction of p42/44 phosphorylation, which in turn abrogated the cytosolic mitogen‐activated protein kinase (MAPK) signaling that is critical for meiosis and mitosis. Saha et al. (2016) suggested that these same growth factors inhibited by AuNPs are also responsible for the disruption in bidirectional crosstalk between pancreatic cancer cells and pancreatic stellate cells as well as MAPK signaling inhibition and EMT reversal in pancreatic ductal adenocarcinoma. AuNPs have also shown to downregulate the levels of other pro‐angiogenic factors such as Ang‐1 and Ang‐2, leading to the induction of p53 and p21 as well as down‐regulation of metastasis‐associated markers, CD44, and CD133 (Satapathy et al., 2018).

AgNPs of various sizes (500 and 16.5 nm) have also been shown to inhibit angiogenesis via the inactivation of the PI3K/Akt signaling pathway (Baharara, Namvar, Mousavi, Ramezani, & Mohamad, 2014; Gurunathan et al., 2009; Kalishwaralal et al., 2009). Other NMs that have shown potential in anti‐angiogenic activity via binding of growth factors include cerium oxide NPs (Giri et al., 2013), graphite, multi‐walled CNTs, fullerenes (Jiao et al., 2010; Meng et al., 2010; Murugesan, Mousa, O'Connor, Lincoln, & Linhardt, 2007), and graphene oxide (Lai et al., 2016). In addition, titanium dioxide (TiO_2_) NPs have been shown to inhibit EMT by interacting with the TGFβ receptor TβRI/II complex and downregulating the expression of TGFβ target genes (X. Li, Song, et al., 2018).

### 2.7 Exosomes

Recently, it has been suggested that NMs could prevent tumor exosome biogenesis in cancer cells, which would thereby prevent their release into the extracellular space. In addition, NMs could further prevent the uptake of already‐released exosomes by healthy secondary cells. Tumor exosomes are small lipid bilayer vesicles released by cancer cells containing proteins, mRNAs, and miRNAs as well as metastasis or invasive‐related molecules, and so on (D. Gao & Jiang, 2018) play an important role in tumor cell communication, TME maturation, and angiogenesis (D. Gao & Jiang, 2018; Roma‐Rodrigues, Fernandes, & Baptista, 2014). The release of tumor exosomes is also induced in stress situations for example, hypoxia, starvation, high acidity, which are common in the TME and which therefore contribute to tumor growth (Roma‐Rodrigues et al., 2014; Villarroya‐Beltri, Baixauli, Gutiérrez‐Vázquez, Sánchez‐Madrid, & Mittelbrunn, 2014). Roma‐Rodrigues, Raposo, et al. (2017) could show a decrease in exosome release simply by incubating MCF‐7 and MDA‐MB453 cells with basic PEGylated AuNPs and suggested that this may be due to the interference of NP internalization with exosome secretion since AuNPs were found to saturate late endosomes that use the same exocytosis pathway as exosomes. This is indeed an important finding worthy of future in‐depth investigation.

### 2.8 Immunotherapy

Immunotherapy addresses one of the major challenges in cancer, which is the immunosuppressive nature of the TME, which limits the immune system's ability to recognize and attack cancer cells (Hess, Medintz, & Jewell, 2019; Y. Huang & Zeng, 2020). NMs themselves may be used to modulate the immunosuppressive TME by targeting mediators that inhibit the immune cycle and that are often the cause of immunotherapy failure for example, tumor‐associated macrophages (TAMs), myeloid‐derived suppressor cells (MDSCs), regulatory T cells (Tregs), and so on. For example, Sacchetti et al. (2013) could successfully target PEGylated SWCNTs to Tregs in a B16 melanoma using ligand‐specific markers against the glucocorticoid‐induced TNFR‐related receptor. Iron‐oxide NPs have also shown to result in the inhibition of both adenocarcinoma growth and liver metastasis in mice through their intrinsic potential to polarize immunosuppressive TAMs into the pro‐inflammatory M1 phenotype (Zanganeh et al., 2016).

The role of pyroptosis in cancer immunotherapy has also recently shown promise. Pyroptosis may be initiated by caspase‐1, caspase‐4/5/11, or caspase‐3 and results in membrane pore formation and cellular swelling (Y.‐Y. Wang, Liu, & Zhao, 2019). Hu et al. (2019) demonstrated that arsenic trioxide (As_2_O_3_) NPs could induce pyroptosis in hepatocellular carcinoma Huh7 and HepG2 cells and inhibit tumor growth in a Huh7 xenograft nude mice model through increased caspase‐3 expression and increased Gasdermin E (GSDME) cleavage by caspase‐3 to produce GSDME N‐fragments. These fragments then enter the cell membrane to induce pore formation and pyroptosis. Similarly, Song, Du, Feng, Wu, and Yan (2013) demonstrated the induction of pyroptosis by ZnO NPs via the caspase‐1 pathway in A549 lung cancer cells. The importance of NM‐induced pyroptosis in immunotherapy is scarce and it has been suggested that the toxicological mechanisms of NMs involving LMP, inflammasome activation, and pyroptosis be considered as a potential research area in cancer therapy (Gulumian & Andraos, 2018). Indeed, LMP could activate pyroptosis via caspase‐1 and NLRP3 inflammasome activation (Repnik, Česen, & Turk, 2014).

Table 1 summarizes the studies discussed above in which the intrinsic abilities of NMs have been used for a specific cellular outcome. Although self‐therapeutic NMs show great promise in cancer nanomedicine, it is still debatable as to whether they exhibit the potency needed for successful cancer eradication in the clinical setting. Often, this potency is only obtainable using externally applied radiation, as discussed in the next section.

**TABLE 1:** NMs exhibiting inherent anti‐cancer properties lead to different mechanistic outcomes depending on the cellular pathway

Outcome	Cellular structure: pathway	NM	Reference
Apoptosis	Nucleus: DSBs, cytokinesis arrest	30 nm PEGylated NLS‐AuNPs	Kang et al., 2010
Apoptosis	Nucleus: Nuclear envelope deformation, increases in caspase‐3 and ‐7 activities, DSBs	32 nm AuNSs nanoconstruct with nucleolin DNA aptamer	Dam, Culver, et al., 2012; Dam, Lee, et al., 2012
LMP followed by apoptosis	Lysosome: Aggregation of NP assemblies and crystals inside lysosomes, lysosomal swelling, LMP	Mixed‐charge 5 nm AuNPs	Borkowska et al., 2020
LMP followed by apoptosis and necrosis	Lysosome: Fe^3+^ burst within lysosomes	28.8 nm Si‐modified zero‐valent iron NPs	L.‐X. Yang et al., 2019
Autophagy followed by apoptosis	Initiate LMP and autophagosome accumulation, mitochondrial function inhibition via ATP production decrease, inhibit mTOR activation, activate ULK1, disrupt both ER and Golgi, ER stress induction	Fe_3_O_4_ NPs	X. Zhang et al., 2016
LMP followed by apoptosis	Lysosome: ROS generation in lysosomes	Au‐ZnO nanohybrid	W. Gao et al., 2014
Apoptosis	Increased LMP and autophagy induction via enhanced LC3B expression, NLRP3‐inflammasome activation via enhanced expression of caspase‐1, IL‐1β, caspase‐3, ER stress induction by GADD153 expression	10–100 nm PVP‐coated AgNPs	Mishra et al., 2016
Autophagy followed by apoptosis	ROS generation and oxidative stress, loss in mitochondrial membrane potential, DSBs, increased expression in p53, LC3, Bax, and caspase‐9, decreased expression of Bcl‐2	20 nm ZnO NPs	Bai et al., 2017
Apoptosis	Accumulation in mitochondria and nucleus, ER stress induction, oxidative stress, and mitochondrial impairment	AgNPs	Buttacavoli et al., 2018
Apoptosis and necrosis	Golgi/ER: ER stress induction, ER vacuolization. Increase in ER stress proteins GRP78 and GADD153	5.48 nm A_S4_S_4_ QDs	H. Wang et al., 2015
Autophagy followed by ferroptosis	Mitochondria: Fenton reaction, ROS production, mitochondrial lipid peroxidation, and reduced levels of GPX4	123.4 nm zero‐valent iron NPs	K.‐J. Huang et al., 2019
Ferroptosis	Mitochondria: Increased iron adsorption and uptake, increased ROS, GSH depletion	6 nm PEGylated SiNPs	Kim et al., 2016
Ferroptosis	Mitochondria: GSH oxidization and depletion, transform endogenous H_2_O_2_ produced by the mitochondria into hydroxyl radicals via Fenton reaction	PEGylated MnO_2_‐coated thiol‐functionalized mesoporous SiNPs	L. S. Lin, Song, et al., 2018
Ferroptosis	Mitochondria: GSH oxidization and depletion, decrease GPX4 activity and expression, oxidative stress, lipid peroxidation	59 nm arginine‐rich manganese silicate nanobubbles	S. Wang, Li, Qiao, et al. (2018)
LMP	Lysosome: Alkalization of lysosomal interior	AuNPs and AgNPs	X. Ma et al., 2011; Miyayama et al., 2018
Autophagic flux blockade	Lysosome: TFEB expression reduction, lysosomal dysfunction, autophagic flux blockade, and autophagosomes accumulation	60 nm AgNPs	Miyayama et al., 2018
Autophagic flux blockade	Autophagolysosomes: Perturbation of autophagy‐mediated protein turnover, degradation of internalized epidermal growth factor, impaired cargo delivery via autophagosomes or late endosomes to SiNP‐filled lysosomes	SiNPs	Schütz et al., 2016
Autophagy	Golgi/ER: Increase LC3‐I to LC3‐II	86 nm SiNPs	Wei et al., 2017
Autophagy	Golgi/ER: ROS generation, ER stress induction via EIF2AK3, and ATF6 pathway activation	57.7 nm SiNPs	J. Wang, Li, Duan, et al., 2018
Angiogenesis inhibition	Bind HB–GFs such as VEGF165 and bFGF and unfold protein structure	5 nm AuNPs	Arvizo et al., 2011; Arvizo et al., 2013; Mukherjee et al., 2005; R Bhattacharya et al., 2004; Mukherjee et al., 2005
Angiogenesis inhibition	Bind VEGF165, prevent interaction with cell surface receptor VEGFR‐2, inhibit receptors phosphorylation	AuNPs	Resham Bhattacharya & Mukherjee, 2008
Angiogenesis inhibition	Cell cycle arrest by up‐regulating p21 and p27 as downstream consequence of AuNPs binding to VEGF	AuNPs	Resham Bhattacharya et al., 2007
Angiogenesis inhibition	Ang‐1 and Ang‐2 downregulation, p53 and p21 induction, downregulation of metastasis‐associated markers	AuNPs	Satapathy et al., 2018
Angiogenesis inhibition	Inactivation of PI3K/Akt signaling pathway	500 and 16.5 nm AgNPs	Baharara et al., 2014; Gurunathan et al., 2009; Kalishwaralal et al., 2009
Angiogenesis inhibition	Binding of angiogenesis‐related growth factors	CeO NPs, graphite, MWCNTs, fullerenes, graphene oxide	Giri et al., 2013; Jiao et al., 2010; Meng et al., 2010; Murugesan et al., 2007; Lai et al., 2016
EMT inhibition	Interact with TGFβ receptor TβRI/II complex and downregulate expression of TGFβ target genes	TiO_2_ NPs	X. Li, Song, et al., 2018
EMT inhibition	Up‐regulating E‐Cad, and down‐regulating Snail, N‐Cadherin, and Vimentin	20 nm AuNPs	Arvizo et al., 2013
Tumor exosome secretion interference	Saturate late endosomes that use the same exocytosis pathway as exosomes	PEGylated AuNPs	Roma‐Rodrigues, Raposo, et al., 2017
Immunotherapy	Target Tregs	PEGylated SWCNTs	Sacchetti et al., 2013
Immunotherapy	Polarize immunosuppressive TAMs into M1	Iron‐oxide NPs	Zanganeh et al., 2016
Pyroptosis	Caspase‐3/Gasdermin E pathway	As_2_O_3_ NPs	Hu et al., 2019
Pyroptosis	Caspase‐1 pathway	ZnO NPs	Song et al., 2013

## 3 NMs LEADING TO LOCALIZATION OF RADIATION FOR PTT‐, PDT‐, OR RADIO‐ENHANCEMENT

NMs may exhibit cell‐killing abilities in the presence of external radiation during which the NM may either produce ROS, heat, or enhance the effect of radiation through localization to a specific cellular structure. PDT involves the interaction between radiation light and a photosensitizing agent (the NM) at a wavelength within the NM's surface plasmon resonance (SPR). This interaction results in the production of free radicals and ROS or singlet oxygen (^1^O_2_) (Calixto, Bernegossi, de Freitas, Fontana, & Chorilli, 2016; Casais‐Molina, Cab, Canto, Medina, & Tapia, 2018; Kwiatkowski et al., 2018; Yoo & Ha, 2012). In PTT, the light couples with the oscillation frequency of conduction electrons within the NM's SPR, resulting in a localized photo‐to‐heat conversion (Boca et al., 2011; Caputo, De Nicola, & Ghibelli, 2014). NMs may also sensitize tumors to radiation via *biological* mechanisms or *physical* mechanisms. The biological mechanisms include those in which NMs could sensitize tumors to radiation via oxidative stress, DNA damage and cell cycle effects among others. The physical mechanisms include those in which NMs directly enhance the efficacy of radiation either by localization or by increasing the intensity of radiation via the Compton, Photoelectric, or Auger effects (Hainfeld, Dilmanian, Slatkin, & Smilowitz, 2008; Haume et al., 2016; Rosa, Connolly, Schettino, Butterworth, & Prise, 2017).

The main advantage of using NMs together with radiation is, as stated above, the enhanced localization of the effect to a specific cellular structure. PTT or PDT applied in the absence of NMs produce a transient, localized effect. The efficacy of these phototherapies is compromised if this localized effect is not directed to a particular cellular site where it can induce enhanced destruction of the cancer cell. Therefore, targeted NMs are often utilized to localize the effect of phototherapies to a particular cellular organelle for guaranteed destruction of the cancer cell.

For NMs to be successful in phototherapies, certain characteristics are desired. For PTT or PDT enhancement, a NM with a strong and tunable localized SPR in the near infrared (NIR) region and with uniform size should be considered. This will allow the selection of a very narrow absorption peak for effective optical irradiation (Xia et al., 2017; F. Zhou et al., 2011; F. Zhou et al., 2010). Shape also directly affects the SPR characteristics and heat conversion abilities of NMs. Gold nanostars (AuNSs) are popular in PTT due to their anisotropic shape that contains multiple sharp pinpoints, which can dramatically enhance the SPR for efficient photothermal transduction (Xia et al., 2017). Other NMs popular in PTT include SWCNTs, AgNPs and additional Au nanostructures including spheres, AuNRs and nanocages (Xia et al., 2017). Copper sulphide (CuS) NPs were also considered an optimal candidate for PTT due to their outstanding NIR optical absorption and high photothermal conversion efficiency (N. Li, Sun, et al., 2018). If radio enhancement is desired, then metals with high‐atomic numbers (i.e., high Z‐NPs) are required. Therefore, Au is by far the most popular radio enhancer due to its high atomic number (*Z* = 79) and mass energy coefficient relative to soft tissue allowing increased energy deposition at the target site (Hubbell & Seltzer, 1995; Rosa et al., 2017). Bismuth (Bi) has also shown great promise in radio enhancement due to its high atomic number (*Z* = 83) (Deng et al., 2018). Although several other elements exhibit high atomic numbers, for example (gadolinium very few of these have been shown to be nontoxic. The following sections discuss the cellular mechanisms in relation to their intracellular organelle localization, which are involved in NMs to localization of radiation for PTT‐, PDT‐, or radio‐enhancement.

### 3.1 Nucleus

Several NMs have been shown to induce nucleolar damage with the assistance of external radiation. AuNPs and Au nanoflowers increased the efficacy of PTT in MCF‐7 cancer cells through the reorganization of the nuclear laminae and envelopes (Kodiha et al., 2014). Platinum NPs and AuNPs have also shown to increase DSBs by localizing applied radiation directly to the DNA strand (Porcel et al., 2010). Anas et al. (2008) showed that streptavidin‐functionalized CdSe‐ZnS QDs complexed with biotinylated plasmid DNA (pDNA) enhanced PDT by inducing DSBs in the pDNA via the generation of ^1^O_2_. A different type of mechanism leading to DSBs by AuNRs have been reported by (Limin Pan et al., 2017). They showed that PEGylated 10.5 × 40.5 nm AuNRs liganded with an NLS resulted in apoptosis via DSBs during PTT by silencing the expression of the DNA replication protein A 70 (RPA70), which is important for single‐stranded DNA binding, replication and repair as well as recombination and damage‐response signaling. Whether this was due to interference with the transcriptional machinery of the protein or direct binding of the protein to the AuNRs is unknown. Mesoporous silica‐coated CuS NPs (40 nm) liganded with an NLS also induced DSBs via PTT in a HeLa tumor mice model (N. Li, Sun, et al., 2018). The mechanism by which DSBs occurred was assumed to occur through direct heat applied to the DNA. Finally, Deng et al. (2018) showed that 56 nm, anionic, folate‐inserted, red blood cell membrane‐modified BiNPs were able to enhance the X‐ray radiation efficacy by increasing the ROS production within 4T1 breast tumor cells leading to an increase in DSBs, although the NPs were not proven to enter the nucleus. Zheng, Yang, Wei, Tong, and Shu (2013) have shown that AuNPs and AgNPs sensitized the response of hepatocellular carcinoma HepG2 cells to radiation through a biological mechanism that is, via DSB‐induction. The exact mechanism leading to DSBs is unknown; however, the authors speculate that oxidative damage was involved as the expression of the antioxidants catalase (CAT), superoxide dismutase (SOD), and glutathione (GSH) was reduced. In addition, the NPs appeared to upregulate the expression of Bax and caspase‐3 and downregulate the expression of the anti‐apoptotic protein Bcl‐2, thereby inducing apoptosis.

Gold NMs could also enhance the radiosensitivity of cancer cells by disruption of cell division (Rosa et al., 2017). For example, Roa et al. (2009) showed that 10.8 nm glucose‐capped AuNPs enhanced the radiosensitivity of human prostate carcinoma DU‐145 cells by accelerating the G1/S phase and arresting the G2/M phase through the activation of checkpoint kinases, CDK1, and CDK2. Roa et al. (2009) suggested that this mechanism directly affects the radiosensitivity of the cell since G2/M is the phase most sensitive to radiotherapy. The AuNPs were mostly distributed in the cytoplasm and not the nucleus so it is not known how the NPs affect the checkpoint kinases. Similarly, Geng et al. (2011) observed cell cycle arrest at the G2/M phase and enhanced radiosensitivity in SK‐OV‐3 ovarian cancer cells treated with 14 nm thio‐glucose bound AuNPs.

### 3.2 Mitochondria

It is well known that mitochondria are highly sensitive to heat, thereby presenting excellent targets for PTT (Lin, Bao, et al., 2018). Ma et al. (2018) used 15.7 nm TPP‐AuNPs to enhance PTT via local heat production at the mitochondria of HeLa cells and 4T1 tumor‐bearing mice resulting in mitochondrial destruction, cytochrome‐C release, caspase‐3 activation, and tumor‐specific apoptosis. Jung et al. (2015) showed that Mito‐CIO consisting of 15 nm TPP‐Fe_3_O_4_ NPs labeled with a coumarin fluorophore for intracellular tracking was able to enhance PTT by directly localizing heat to the mitochondria of HeLa cells, which induced both apoptosis and necrosis. CNTs have also shown promise in mitochondrial‐targeted PTT. For example, Zhou et al. (2011) and Zhou et al. (2010) showed that PEGylated SWCNTs (0.81 nm × 500–1,500 nm) was able to localize PTT within mitochondria, thereby inducing mitochondrial depolarization, cytochrome‐C release, caspase‐3 activation, and apoptosis. These caspases can, in return, disturb the mitochondrial electron transport chain and induce a domino effect on ROS burst, which causes irreversible cell death.

As mitochondria are also highly sensitive to ROS imbalance, it is only evident that it would enhance PDT. For example, Yu, Sun, Pan, Li, and Tang (2015) developed a NIR‐triggered nano‐photosensitizer consisting of TPP‐TiO_2_‐coated, NaYF_4_ upconversion NPs, which converts low energy NIR radiations to high energy visible radiations. When irradiated in MCF‐7 cells and in an MCF‐7 mice model, this nano‐photosensitizer produced ROS in mitochondria thereby initiating the intrinsic apoptotic pathway. Apoptosis was accompanied with the activation of an inner membrane anion channel (IMAC), the opening of mitochondrial permeability transition pores, the decrease in mitochondrial membrane potential, cytochrome‐C release, and caspase‐3 and ‐7 activation.

### 3.3 Lysosome, autophagosome, and autophagolysosome

The Fe_3_O_4_ NPs appear to be popular in LMP induction of cancer cells as these NPs allow controlled LMP via sheer mechanical force to the lysosomal membrane in the presence of a magnetic field. For example, Zhang et al. (2014) showed that superparamagnetic Fe_3_O_4_ NPs targeted to the lysosomal protein marker, LAMP1, via LAMP1 antibodies was able to induce apoptosis in both rat insulinoma tumor cells and human pancreatic beta cells via LMP simply by creating shear forces through the generation of oscillatory torques with the application of a magnetic field. Domenech, Marrero‐Berrios, Torres‐Lugo, and Rinaldi (2013) also demonstrated the use of Fe_3_O_4_ NPs in inducing LMP in MDA‐MB‐231 breast cancer cells upon an applied magnetic field; however, their NPs accumulated within the lysosomes without any need for targeting and the authors suspected that ROS generation and LMP might have been induced via mechanical sheering and/or heat. Moosavi et al. (2016) showed that nitrogen‐doped TiO_2_ NPs enhanced PDT in K562 leukemia cells through ROS generation and autophagy induction during which LC3 was cleaved by autophagy‐related protein 4 (Atg4) and subsequently conjugated to phosphatidylethanolamine (LC3‐II) by Atg3, which allowed LC3‐II to insert into autophagosomes membranes. The authors also observed the occurrence of autophagy flux through the detection of the lysosomal marker LAMP2, which co‐localized with cytosolic LC3‐II. The authors showed that induction of autophagy led to the intrinsic apoptotic pathway via activation of caspase‐9. Zhang et al. (2017) constructed a PEGylated Ru(II) (RuPEG) loaded onto a reduced graphene oxide sheet (rGO). The nanohybrid entered the lysosomes of human lung cancer A549 cells during which the rGO was released from RuPEG. Upon irradiation, the RuPEG induced PDT while the rGO induced PTT. The combined PDT and PTT treatment resulted in lysosomal damage and consequently apoptosis through ROS generation.

### 3.4 Golgi/ER

Literature addressing the NM‐induced destruction of the Golgi/ER complex in the presence of radiation is lacking. However, Chang et al. (2008) has demonstrated the potential of 13 nm AuNPs in improving the outcome of cancer radiotherapy in B16F10 melanoma cells and a B16F10 melanoma mice model through co‐localization of the AuNPs with the ER/Golgi complex and the induction of apoptosis. Similarly, Mocan et al. (2015) showed that 49 nm albumin‐coated AuNPs in HepG2 cells initiated the disintegration of the Golgi/ER complex following PTT, which ultimately led to the activation of caspase‐3 and apoptosis.

### 3.5 Cell membrane

Huang, Delikanli, Zeng, Ferkey, and Pralle (2010) targeted 6 nm superparamagnetic manganese ferrite (MnFe_2_O_4_) NPs to cells expressing the temperature‐sensitive transient receptor potential vanilloid 1 (TRPV1) ion channel. The TRPV1 is a member of the TRP family, which has been identified in cortical and subcortical areas of the brain (Rosenbaum & Simon, 2006). These NPs enhanced PTT through heat, which led to the opening of the TRPV1 channels resulting in an influx of calcium ions (Ca^2+^) and cell depolarization. An apoptotic cell death mechanism was suspected due to the rapid Ca^2+^ influx as previous studies have demonstrated (Ramirez et al., 2018).

### 3.6 Immunotherapy

NMs may target cancer cells through enhanced radiation localization as well as simultaneously initiate host immune responses thereby also enhancing cancer immunotherapy. For example, Bear et al. (2013) showed the systemic effects of PTT in the presence of Au nanoshells in a melanoma model and observed that PTT promoted a tumor‐specific immune response against a distant, subcutaneous B16‐ovalbumin tumor. This response was mediated by the infiltration of CD4+ helper T cells and CD8+ cytotoxic T cells. PTT was followed by a systemic increase in inflammatory cytokines such as IL‐6 and IL‐1β, as well as an increase in factors such as granulocyte‐(macrophage) colony‐stimulating factors (G(M)‐CSFs). Similarly, Nguyen, Tran, Sun, and Shen (2012) could show that Au nanoshell/silica core NPs following PTT could induce necrosis in TC1 cells as well as the release of damage associated molecular patterns (DAMPs). Table 2 shows a summary of the studies discussed above in which NMs in the presence of radiation have been used for a specific mechanistic outcome.

**TABLE 2:** NMs leading to localization of external radiation for PTT‐, PDT‐, or radio‐sensitization

Outcome	Cellular structure: Pathway	NM	Reference
PTT enhancement	Nucleus: Nuclear laminae and envelope reorganization, nucleolar function inhibition	AuNPs and Au nanoflowers	Kodiha et al., 2014
Radiosensitization	Nucleus: DSBs	Gold and platinum NPs	Porcel et al., 2010
PDT enhancement	Nucleus: DSBs, ROS generation	CdSe‐ZnS QDs	Anas et al., 2008
PTT enhancement via apoptosis	Nucleus: DSBs via silencing expression of DNA replication protein A 70	10.5 × 40.5 nm PEGylated NLS‐AuNRs	Limin Pan et al., 2017
PTT enhancement	Nucleus: DSBs via direct heat localized to DNA	40 nm mesoporous silica‐coated CuS NPs	N. Li, Sun, et al., 2018
Radiosensitization	Nucleus: DSBs via ROS production by localization of radiation	56 nm red blood cell membrane‐modified BiNPs	Deng et al., 2018
Apoptosis and radio sensitization	Nucleus: DSBs, oxidative damage, reduction of CAT, SOD and GSH expression, upregulation of Bax and caspase‐3 expression and down‐regulation of Bcl‐2	AuNPs and AgNPs	Zheng et al., 2013
Radiosensitization	Nucleus: Accelerate G1/S phase and arrest G2/M phase via CDK1 and CDK2 activation	10.8 nm glucose‐capped AuNPs	Roa et al., 2009
Radiosensitization	Nucleus: Cell cycle arrest at G2/M phase	14 nm thio‐glucose AuNPs	Geng et al., 2011
PTT enhancement via apoptosis	Mitochondria: Direct heat localized to mitochondria, cytochrome‐C release, caspase‐3 activation	15.7 nm TPP‐AuNPs	Z. Ma et al., 2018
PTT enhancement via apoptosis and necrosis.	Mitochondria: Direct heat localized to mitochondria	15 nm TPP‐Fe_3_O_4_ NPs	Jung et al., 2015
PTT enhancement via apoptosis	Mitochondria: Direct heat localized to mitochondria, induce mitochondrial depolarization, cytochrome‐C release, caspase‐3 activation, disturbance in mitochondrial electron transport chain, and domino effect on ROS burst	0.81 nm × 500–1,500 nm PEGylated SWCNTs	F. Zhou et al., 2011; F. Zhou et al., 2010
PDT enhancement via apoptosis	Mitochondria: Direct ROS localized to mitochondria, IMAC activation, opening of transition pores, mitochondrial membrane potential decrease, cytochrome‐C release, caspase‐3 and ‐7 activation	TPP‐TiO_2_‐coated NaYF_4_ upconversion NPs	Yu et al., 2015
Apoptosis via LMP by magnetic field	Lysosomes: Create shear forces through the generation of oscillatory torques via magnetic field	Fe_3_O_4_ NPs targeted to LAMP1, via LAMP1 antibodies	E. Zhang et al., 2014
LMP by magnetic field	Lysosomes: ROS generation and LMP via mechanical sheering and/or heat	Fe_3_O_4_ NPs	Domenech et al., 2013
PDT enhancement via autophagy and apoptosis	ROS generation, LC3 cleaved by Atg4 and conjugated to phosphatidylethanolamine by Atg3, which allowed LC3‐II to insert into autophagosome membranes. Autophagy induction, caspase‐9 activation, apoptosis	Nitrogen‐doped TiO_2_ NPs	Moosavi et al., 2016
PDT and PTT enhancement via apoptosis	Lysosomes: RuPEG induced PDT, rGO induced PTT, ROS generation, lysosomal damage	PEGylated Ru(II)‐rGO nanohybrid	D.‐Y. Zhang et al., 2017
Radiosensitization, apoptosis	Golgi/ER: Direct radiation localized to ER and Golgi	13 nm AuNPs	Chang et al., 2008
PTT enhancement via apoptosis	Golgi/ER: Disintegration of Golgi/ER complex, caspase‐3 activation	49 nm albumin‐coated AuNPs	Mocan et al., 2015
PTT enhancement via apoptosis	Cell membrane: Direct heat localization, opening of TRPV1 channels, Ca^2+^ influx, cell depolarization	6 nm MnFe_2_O_4_ NPs	H. Huang et al., 2010
PTT, immunotherapy	Infiltration of CD4+ helper T cells and CD8+ cytotoxic T cells, systemic increase in inflammatory cytokines IL‐6 and IL‐1β, increase in GM‐CSF and G‐CSF.	Au nanoshells	Bear et al., 2013
PTT, immunotherapy	Necrosis and release of DAMPs	Au nanoshell/silica core particles	Nguyen et al., 2012

## 4 NMs AS NANOCARRIERS IN THE ABSENCE OR PRESENCE OF RADIATION

NMs as delivery vehicles for therapeutic compounds is perhaps to date the most studied therapeutic strategy in cancer nanomedicine (Hossen et al., 2019; Senapati, Mahanta, Kumar, & Maiti, 2018). It is known that certain compounds become unstable in vivo thereby decreasing their bioavailability (Huo et al., 2014; Pollard et al., 1998; Seynhaeve, Dicheva, Hoving, Koning, & ten Hagen, 2013). In addition, increased adverse side effects are often observed, as selective tumor targeting of conventional chemo drugs is difficult to achieve. Consequently, higher application dosages of the drug are often required, which, in effect, increases the likelihood of adverse side effects. By attaching these chemo drugs to nanocarriers, their bioavailability is enhanced. All NM types including those consisting of metals, metal oxides, silica, and carbon, among others, have successfully served as nanocarriers for the following:

1. Conventional drugs for example, cisplatin, camptothecin, and doxorubicin (DOX).
2. Small interfering RNAs (siRNAs) and triplex‐forming oligonucleotides for gene therapy.
3. Photosensitizers molecules for enhanced phototherapies.
4. Growth factor antibodies (e.g., VEGF antibody), microRNAs (miR‐503), or the anti‐angiogenic drug, sorafenib, for angiogenesis inhibition.
5. For cancer antigens, adjuvants, peptide‐, or DNA‐based vaccines or immune checkpoint modulators for cancer immunotherapy (ClinicalTrials.gov, 2018; Han, Tang, & Yin, 2016; Huo et al., 2014; Jiang, Huo, Hardie, Liang, & Rotello, 2016; Mukherjee et al., 2007; Naz et al., 2019; Limin Pan et al., 2012; L. Pan et al., 2018; Wu et al., 2015).

The most critical characteristic for successful nanocarriers is undoubtedly their pore structures and surface topographies. Mesoporous SiNPs are known for their efficient drug loading capacities (L. Pan et al., 2018) as silica is known for its tunable pore structure and ease of synthesis of various surface topographies (Singh, Knowles, & Kim, 2019). Metal and metal oxide NMs are also useful drug delivery vehicles due to their controllable size and shape and ease of surface functionalization. Certain shapes of NMs are also important for increased surface areas and hence increased drug loading. For example, Dam, Culver, et al. (2012), Dam, Lee, et al. (2012), and Xia et al. (2017) used AuNSs simply for their increased surface to volume ratio allowing increased drug/ligand loading and surface modification.

The role of NMs as successful nanocarriers have been extensively reviewed elsewhere (Naz et al., 2019) and the intracellular mechanisms initiated as well as the organelles targeted would in effect be determined by the drug that is carried by the NM. As a result, this section of the review will not be sub‐divided by organelle type as with Section 1 and 2. However, this section will briefly summarize how nanocarriers could potentiate the effects of therapeutic compounds, how nanocarriers aid in overcoming MDR, the role of exosomes in nanocarrier therapy and, last, how nanocarriers themselves could serve as adjuvants while carrying antigens and/or adjuvants to the tumor site.

Not only may nanocarriers successfully deliver therapeutic compounds, but they may also potentiate the effect of the compound being delivered. For example, a 6.6 nm Fe_3_O_4_/Gd_2_O_3_ NP nanocarrier successfully delivered and potentiated the effect of cisplatin in orthotopic glioblastoma cells. The Fe^2+^ and Fe^3+^ released from the nanocarrier directly participated in the Fenton reaction and ROS production, while the cisplatin indirectly produced H_2_O_2_, thereby further accelerating the Fenton reaction and inducing ferroptosis (Shen, Liu, et al., 2018). A TPP‐AuNP nanocarrier successfully delivered the energy‐blocker 3‐ bromopyruvate (BP) to mitochondria of cancer cells. The 3‐BP demolished mitochondrial oxidative phosphorylation and upon irradiation, the AuNPs could further induce mitochondrial destruction via PTT resulting in apoptosis (Marrache & Dhar, 2015). A reduced graphene oxide (rGO)–AgNPs nanocomposite in combination with cisplatin resulted in more pronounced effects on the expression of autophagy genes and in the accumulation of autophagosomes and autophagolysosomes in HeLa cancer cells, which were associated with ROS generation and apoptosis (Yuan & Gurunathan, 2017). Similarly, the combination of the drug, salymicin, and AgNPs showed a substantial synergistic effect on cytotoxicity and in the accumulation of autophagolysosomes in A2780 ovarian cancer cells. The induction of massive autophagy, in turn, led to mitochondrial dysfunction and cell death via apoptosis (X.‐F. Zhang & Gurunathan, 2016). Recently, Pool et al. (2018) demonstrated the anti‐proliferative effects of PEGylated silica NMs carrying the drug, genistein, which activated apoptosis and autophagy in HT29 human colon cancer cells via modulation of antioxidant enzymatic activity (SOD and CAT) and oxidative stress level.

Nanocarriers may also overcome (MDR in cancer cells. For example, Xia et al. (2017), Tu et al. (2018) and Gopisetty et al. (2019) designed nanocarriers consisting of AuNSs, nanographene sheets and AgNPs, respectively, carrying DOX. The nanocarriers consisting of AuNS and nanographene sheets destroyed the mitochondria via PTT, which resulted in the lack of ATP needed for the P‐glycoprotein (P‐gP) pumping system to pump DOX out of the cell before it reached the nucleus (Tu et al., 2018; Xia et al., 2017). The nanocarrier consisting of AgNPs induced ER stress in MDR MCF‐7/KCR cells by inducing the expression of ER stress markers (Grp94, Grp78/Bip, and GADD153) and depleting ER calcium levels. This resulted in a decrease in the number of properly folded P‐gP reaching the plasma membrane and, as a result, allowed DOX to enter the nucleus and induce apoptosis. Therefore, all three of these systems successfully inhibited MDR and potentiated the effect of DOX. The disadvantage of nanocarriers is undoubtedly their large size, which could be addressed by the design of stimulus‐triggered nanocarriers. For example, Qiu et al. (2015) constructed a nanocarrier system that could change its size as well as overcome MDR of cancer cells. Their large 60 nm DOX‐loaded assemblies consisting of AgNPs and Ag–AuNRs increased EPR and cellular internalization. However, once inside the cell, DNA dehybridization was initiated via NIR irradiation, which allowed the release of the DOX‐loaded NPs from the NR and subsequent internalization of the DOX into the nucleus of MDR cells.

The role of exosomes in nanocarrier therapy have also recently gained attention. Tumor exosomes may be used as a lipid bilayer to enclose nanocarriers (biomimetic NMs) in order to decrease their toxicity and increase their uptake into tumor tissue (Li, Yang, et al., 2018; Huiping Sun et al., 2017; Yong et al., 2019). Nanocarriers may also be used to decrease tumor exosome formation by delivering oligonucleotides for the silencing of genes involved in exosome biogenesis and secretion. Using AuNPs functionalized with the oligonucleotides anti‐RAB27A, Roma‐Rodrigues, Pereira, et al. (2017) showed the successful silencing of the *RAB27A* gene, which encodes the Rab27a protein involved in vesicle trafficking, budding, mobility, docking at the plasma membrane and fusion. The authors could show successful decrease of exosome release in both MCF‐7 and MDA‐MB453 cells when treated with the functionalized AuNPs. In addition, the few exosomes that were released from AuNP‐treated MCF‐7 cells showed genetic alterations within healthy Bronchial/Tracheal epithelial secondary cells, thereby suggesting that communication between cancer cells and secondary cells could be altered. Roma‐Rodrigues, Raposo, et al. (2017), further suggested using AuNPs as nanovectors for proteins, miRNAs, siRNAs, antisense hairpins, and peptides to directly target molecules that are involved in exosome uptake such as actin cytoskeleton, PI3K, and dynamin 2, to prevent the uptake of tumor exosomes by normal secondary cells. These suggestions indeed allow for numerous new possibilities in which NMs may be used for successful tumor inhibition by targeting the tumor cell secretome.

Although NMs may successfully serve as nanocarriers for antigens and/or adjuvants in immunotherapy, NMs themselves may serve as “self‐adjuvants” to further stimulate an immune response while carrying antigens. For example, 23 nm AuNPs could successfully serve as nanocarriers in a mice tumor model for a red fluorescent protein (RFP) antigen and a CpG 1668 oligodeoxynucleotide adjuvant known to strongly stimulate immune responses through activation of toll‐like receptor 9 (TLR9) (Lee et al., 2012). However, these AuNPs could also facilitate T cell proliferation in an antigen‐specific manner in the absence of the CpG 1668 adjuvant, thereby demonstrating their “self‐adjuvant” ability The “self‐adjuvant” ability of AuNPs were also observed in the studies of Bastús et al. (2009) and Almeida, Lin, Figueroa, Foster, and Drezek (2015). Alizadeh et al. (2018) could demonstrate that SWCNTs functionalized with the antigen, CpG, selectively inhibited the migration of glioma cells and decreased their NF‐κB activation, while activating the immune system, particularly macrophages, by induction of the TLR9/NF‐κB pathway. However, carbon‐based nanotubes and hollow spheres as well as silicon‐based NMs could also demonstrate “self‐adjuvant” abilities in cancer immunotherapy (Fontana et al., 2017; Jambhrunkar et al., 2018; Mahony et al., 2013; Parra, Abad‐Somovilla, Mercader, Taton, & Abad‐Fuentes, 2013).

Nanocarriers have also shown to be effective artificial APCs (aAPCs), by delivering stimulatory signals to activate the immune system. For example, 50–100 nm iron dextran nanocarriers were successful aAPCs by delivering MHC‐Ig dimer and CD28 antibodies (Perica, Bieler, et al., 2014; Perica et al., 2015). The same research group also synthesized two aAPCs consisting of 50–100 nm iron dextran paramagnetic NPs and 30 nm avidin‐coated QDs nanocrystals. Both of these aAPCs contained an MHC‐Ig dimer and biotinylated anti‐CD28 antibody and resulted in effective T‐cell stimulation and inhibition of tumor growth in a subcutaneous mice melanoma model (Perica, Medero, et al., 2014).

Finally, nanocarriers together with externally applied radiation has proven to be successful immunotherapies for certain cancers. Irradiation can induce thermal destruction in tumor cells and the release of cancer antigens can help launch a systemic immune response able to target cancer cells elsewhere in the body (Hess et al., 2019). For example, a 0.8 nm SWCNT nanocarrier delivering the adjuvant, glycated chitosan (GC) could induce PTT‐related tumor cell destruction while GC activated the innate immunity response (F. Zhou et al., 2012). This resulted in complete tumor regression in tumor mice models and in resistance to tumor cell re‐challenge. Similarly, nanocarriers consisting of PEGylated AuNR, AuNPs, or Fe_3_O_4_ delivering adjuvants could effectively kill tumors via PTT and trigger strong immune responses against cancer models. In addition, these nanocarriers could protect against further tumor recurrence (Ge et al., 2018; Yata et al., 2017; B. Zhou et al., 2018). The Fe_3_O_4_ nanocarrier also combined with the checkpoint blockade adjusted by programmed death ligand 1 (PD‐L1), which resulted in the potentiation of the systemic therapeutic efficiency of PD‐L1 checkpoint blockade therapy by activating both innate and adaptive immune systems in the body. Table 3 summarizes the studies discussed above in which nanocarriers have been used for a specific mechanistic outcome.

**TABLE 3:** Nanocarriers resulting in different mechanistic outcomes depending on the cellular pathway

Outcome	Cellular structure: Pathway	NMs	Therapeutic compound delivered	Reference
Ferroptosis by cisplatin potentiation	Enhanced Fenton reaction, ROS production	6.6 nm Fe_3_O_4_/Gd_2_O_3_ NPs	Cisplatin	Shen, Liu, et al., 2018
Apoptosis by 3‐BP potentiation	Mitochondrial destruction via PTT and oxidative phosphorylation	TPP‐AuNP	3‐BP energy‐blocker	Marrache & Dhar, 2015
Apoptosis by cisplatin potentiation	Pronounced of autophagy gene expression, autophagosomes and autophagolysosomes accumulation, and ROS generation	rGO–AgNP nanocomposite	Cisplatin	Yuan & Gurunathan, 2017
Autophagy and apoptosis by salymicin potentiation	Autophagolysosome accumulation and mitochondrial dysfunction	AgNPs	Salymicin	X.‐F. Zhang & Gurunathan, 2016
Apoptosis and autophagy by genistein potentiation	Modulation of antioxidant enzymatic activity and oxidative stress level	PEGylated silica NMs	Genistein	Pool et al., 2018
MDR	Mitochondrial destruction via PTT, lack of ATP needed for P‐gP pumping system	AuNSs	DOX	Xia et al., 2017
MDR	Mitochondrial destruction via PTT, lack of ATP needed for P‐gP pumping system	Nanographene sheets	DOX	Tu et al., 2018
MDR	ER stress induction by Grp94, Grp78/Bip, GADD153 expression, ER calcium depletion. Decrease in number of properly folded P‐gP reaching plasma membrane	AgNPs	DOX	Gopisetty et al., 2019
Decrease in tumor exosome release	Silencing of *RAB27A* gene	AuNPs	Oligonucleotide anti‐RAB27A	Roma‐Rodrigues, Pereira, et al., 2017
Self‐adjuvants in immunotherapy	Increased stimulation of immune response in presence of antigen	23 nm AuNPs; Carbon‐based nanotubes and hollow spheres; Silicon‐based NMs; SWCNTs	Antigens	Lee et al., 2012; Bastús et al., 2009; Almeida et al., 2015; Fontana et al., 2017; Jambhrunkar et al., 2018; Mahony et al., 2013; Parra et al., 2013); Alizadeh et al., 2018
aAPCs in immunotherapy	Deliver stimulatory signals to activate immune system	50–100 nm iron dextran nanocarriers	MHC‐Ig dimer and CD28 antibodies	Perica, Bieler, et al., 2014; Perica et al., 2015
aAPCs in immunotherapy	Deliver stimulatory signals to activate immune system	50–100 nm iron dextran paramagnetic NPs; 30 nm avidin‐coated QDs nanocrystals.	MHC‐Ig dimer and CD28 antibodies	Perica, Medero, et al., 2014
Immunotherapy with radiation	Induce PTT‐related tumor cell destruction of tumor cells while activating immune response (for Fe_3_O_4_ NPs, combined with the checkpoint blockade adjusted by PD‐L1)	0.8 nm SWCNT, PEGylated AuNR, AuNPs, Fe_3_O_4_ NPs	Adjuvant	F. Zhou et al., 2012; Ge et al., 2018; Yata et al., 2017; B. Zhou et al., 2018

## 5 CONCLUSION

In conclusion, a myriad of cellular mechanisms have been exploited in cancer nanomedicine to date, which may roughly be grouped into those NMs exhibiting inherent anti‐cancer properties that is, self‐therapeutic NMs (Group 1), NMs leading to localization of phototherapies or radio‐enhancement (Group 2), and NMs as nanocarriers in the absence or presence of external radiation (Group 3). Understanding the cellular mechanisms, advantages, and physicochemical characteristics of NMs that are important in each of these groups will assist in the selection of the most appropriate strategy and NM design for cancer eradication as well as allow future nanomedicine development.

## CONFLICT OF INTEREST

The authors declare that they have no competing interests.

## AUTHOR CONTRIBUTIONS

**Charlene Andraos:** Conceptualization; data curation; methodology; validation; visualization; writing‐original draft; writing‐review and editing. **Mary Gulumian:** Conceptualization; data curation; funding acquisition; resources; supervision; visualization; writing‐review and editing.

## RELATED WIREs ARTICLES

Physical‐, chemical‐, and biological‐responsive nanomedicine for cancer therapy
